# Successful thrombolytic therapy with recombinant tissue plasminogen activator in ischemic stroke after idarucizumab administration for reversal of dabigatran: a case report

**DOI:** 10.1186/s13256-019-2326-y

**Published:** 2019-12-26

**Authors:** Toshiyuki Ohtani, Ryosuke Sintoku, Tasuku Yajima, Naoyuki Kaneko

**Affiliations:** 1 Department of Neurosurgery, Fukaya Red-Cross Hospital, 5-8-1, Kamishiba-Nishi, Fukaya, Saitama, Japan; 2 Department of Trauma and Emergency, Fukaya Red-Cross Hospital, Saitama, Japan

**Keywords:** Lacunar stroke, Direct oral anticoagulants, Thrombolysis

## Abstract

**Background:**

Idarucizumab is a specific antidote for the anticoagulant dabigatran. Although its efficacy has been recently reported, the drug is still in postmarketing surveillance and requires case data in different emergency settings. A newer intravenous thrombolytic therapy with recombinant tissue plasminogen activator has been proposed after injection of idarucizumab in patients receiving dabigatran; however, the safety and efficacy of this therapy are equivocal because of the limited number of reported cases. We describe a case of a patient with acute lacunar stroke causing dysarthria and hemiparesis successfully treated with intravenous thrombolytic therapy with recombinant tissue plasminogen activator after reversal of dabigatran with idarucizumab.

**Case presentation:**

A 67-year-old Asian woman was transferred to our emergency center 200 minutes after sudden onset of dysarthria and right-sided hemiparesis. She had been taking dabigatran for prevention of stroke recurrence caused by atrial fibrillation. Diffusion-weighted magnetic resonance imaging revealed a new lacunar infarction near old putamen infarctions. We treated her with intravenous thrombolytic therapy with recombinant tissue plasminogen activator after administering idarucizumab. The time to recombinant tissue plasminogen activator administration was 5 minutes from idarucizumab injection and 269 minutes from symptom onset. The patient’s activated partial thromboplastin times were 68.0 and 43.2 seconds before and after the therapy, respectively. The patient’s neurological symptoms improved significantly after the treatment, and she experienced no adverse events.

**Conclusions:**

Intravenous thrombolytic therapy with recombinant tissue plasminogen activator after reversal of dabigatran with idarucizumab may be safe and feasible in patients with acute ischemic stroke with lacunar infarct. Furthermore, intravenous thrombolytic therapy with recombinant tissue plasminogen activator could be used in patients in emergency settings until just before the end of the recommended time limit within which it needs to be administered because of the immediate effect of idarucizumab.

## Background

Although direct oral anticoagulants (DOACs) are widely used for stroke prevention in patients with nonvalvular atrial fibrillation (NVAF), ischemic stroke can still occur in patients undergoing treatment with DOACs. Furthermore, when an ischemic attack occurs, DOACs are associated with a high risk of hemorrhage, especially in patients requiring thrombolytic therapy. Idarucizumab, a humanized monoclonal antibody fragment, is a specific antidote that can reverse the anticoagulant effect of dabigatran, one of the widely used DOACs, rapidly and completely within a few minutes after injection [1]. Although a newer intravenous thrombolytic (IVT) therapy with recombinant tissue plasminogen activator (rt-PA) has been proposed after injection of idarucizumab in patients receiving dabigatran [2, 3], the safety and efficacy of this therapy are equivocal because of the limited number of reported cases. We present a case of a patient with acute lacunar infarct who was successfully treated by IVT therapy with rt-PA after using idarucizumab.

## Case presentation

A 67-year-old Asian woman was transferred to our emergency center 200 minutes after sudden onset of dysarthria and right-sided hemiparesis. She had a history of diabetes mellitus and was receiving antidiabetic medication. She had developed lacunar infarct about 10 years ago with very mild right-sided hemiparesis sequelae and was receiving dabigatran 110 mg twice daily to prevent stroke due to NVAF.

**Fig. 1:**
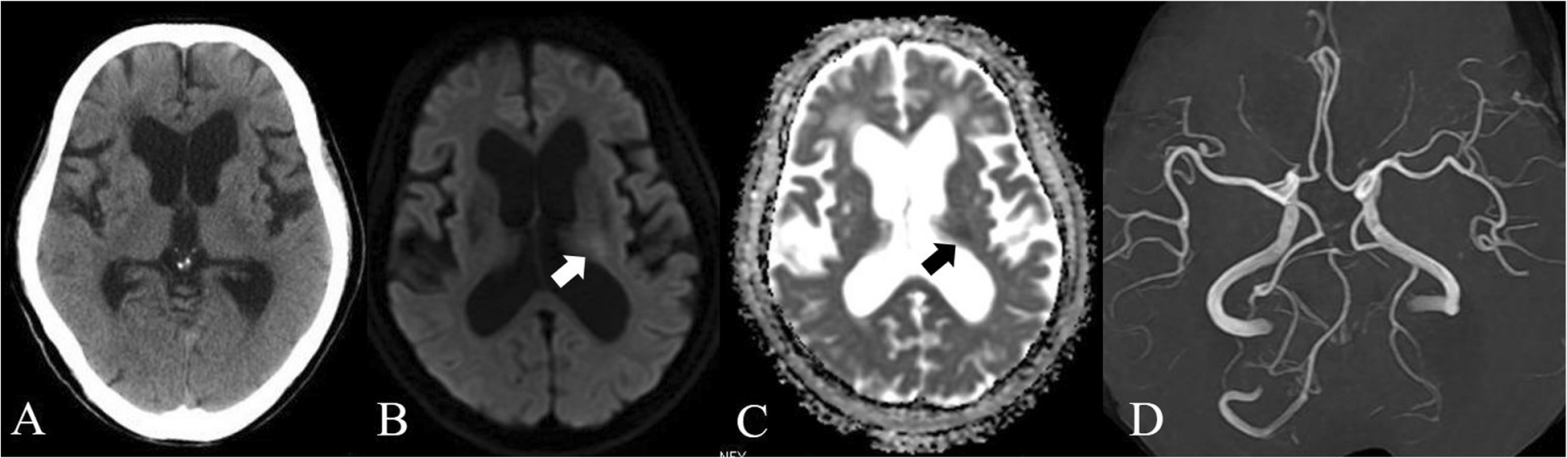
Images imported to our emergency center upon admission. Head computed tomography immediately after our emergency center import and prior to administration of idarucizumab showing a small, low-density spot in the right putamen due to old lacunar infarct, and no cerebral hemorrhage (**a**). Mild high-intensity signals on diffusion-weighted magnetic resonance (MR) imaging revealed the left internal capsule (**b**; *white arrow*) and reduced apparent diffusion coefficient (**c**; *black arrow*). MR angiography showed no large-vessel occlusion (**d**)

The patient’s National Institutes of Health Stroke Scale (NIHSS) score in the emergency room was 7. Computed tomography (CT) of the head was performed at 20 minutes after arrival in the hospital. The scan showed a small, low-density spot in the left putamen, representing the old lacunar infarct (Fig. 1a). Diffusion-weighted magnetic resonance (MR) images revealed a mild hyperintense area in the posterior limb of the left internal capsule, and apparent diffusion coefficient mapping revealed a hypointense area in the region (Fig. 1b and c). Cerebral large vessel occlusion was not detected by MR angiography (Fig. 1d). The patient’s activated partial thromboplastin time (aPTT) was prolonged to 68.0 seconds. The patient and her family were informed regarding the rationale for IVT therapy with rt-PA after using idarucizumab, and they accepted the treatment. An intravenous bolus of 5.0 g of idarucizumab was administered at 264 minutes after the onset of symptoms. Five minutes later, 24 million units of rt-PA were administered intravenously. aPTT just after initiation of the rt-PA infusion was 43.2 seconds.

**Fig. 2:**
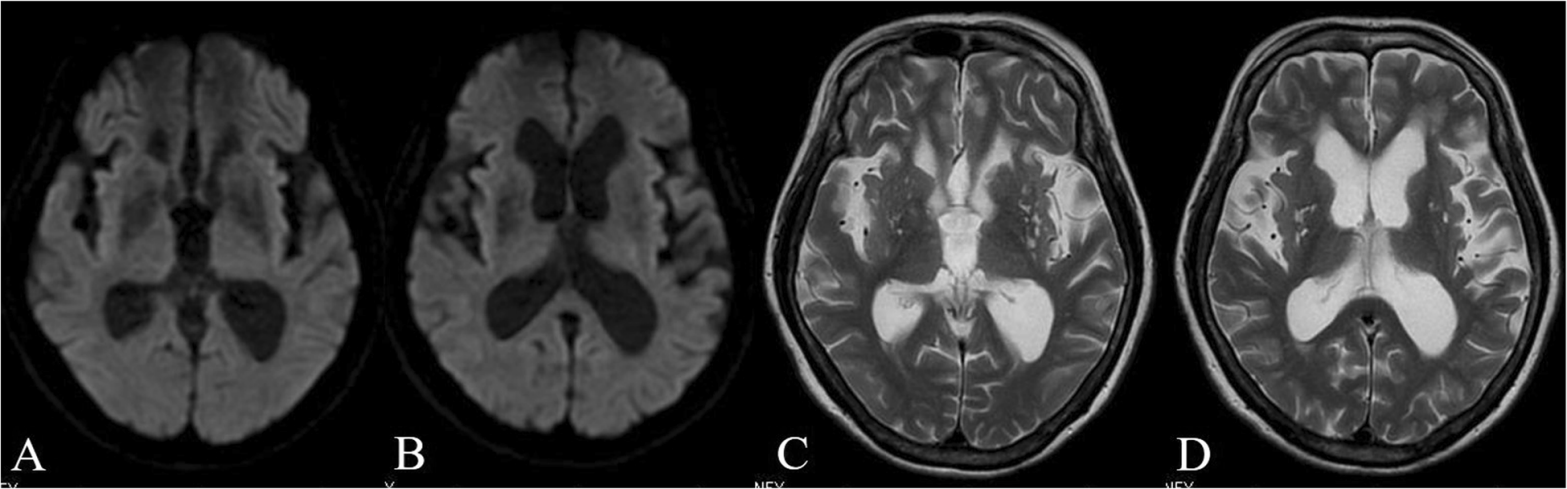
Follow-up magnetic resonance (MR) images on day 4. Diffusion-weighted MR image showing prominent regression of the hyperintense lesion (**a**, **b**). T2-weighted MR image showing small high-intensity spots in bilateral putamen due to old ischemic lesion, but no new abnormality was observed in the posterior limb of the left internal capsule (**c**, **d**)

The patient’s NIHSS score improved from 7 to 4 after 60 minutes of the rt-PA administration. A CT scan the next day showed no hemorrhage. Oral dabigatran administration was resumed 24 hours after IVT therapy. The patient improved neurologically and was ambulatory 3 days later. Diffusion-weighted MR images on day 4 showed that the hyperintense area observed on the initial MR images had disappeared (Fig. 2a and b). Further, there was no new abnormality on the T2-weighted MR images (Fig. 2c and d). The patient did not manifest any systemic thrombotic adverse event due to idarucizumab during the course of treatment. At discharge from the hospital on day 9, her modified Rankin Scale score was grade 2, and her Barthel index was 90 points (reduction of 10 points for bathing and climbing stairs).

## Discussion

We report a case of a patient with acute lacunar stroke causing dysarthria and hemiparesis who was successfully treated by IVT therapy with rt-PA after reversal of dabigatran with idarucizumab.

DOACs have been increasingly used for stroke prevention in patients with NVAF. Dabigatran is one of the major DOACs acting as a direct thrombin inhibitor. Idarucizumab is a humanized monoclonal antibody fragment and is now available as the specific antidote that rapidly neutralizes the anticoagulant effect of dabigatran [4]. IVT therapy with rt-PA has been proposed after using idarucizumab in patients with acute ischemic stroke who are receiving dabigatran [2, 3], and a few articles have reported case studies and systematic reviews [5–7]. In a study that reviewed intravenous thrombolysis in stroke after dabigatran reversal with idarucizumab, 45 of 55 cases benefited from the treatment with a median NIHSS score improvement of 5, whereas unfavorable outcomes were observed in 6 patients (4 deaths, 2 patients with worsening of NIHSS scores, and 1 patient who developed another stroke 30 hours after the first IVT therapy) [7]. In that review, in the available data of 37 patients, 56.76% of patients had grades 0–2 on the modified Rankin Scale at follow-up; hemorrhage extension occurred in 4 patients that ultimately resulted in 1 asymptomatic patient, 1 with severe disability, and 2 deaths. Thus, real-world data seem to indicate that this treatment is safe and effective [7].

During administration of IVT therapy with rt-PA after reversal of dabigatran with idarucizumab, the possibilities of adverse events such as thrombosis and hemorrhage and deterioration in clinical condition need to be borne in mind. Some reports have described thrombotic complications such as deep vein thrombosis [5], pulmonary embolism [5], acute arterial occlusion of the right lower limb [8], recurrence of ischemic stroke [7, 9], and cerebral edema [7, 10]. Furthermore, extension of hemorrhage may cause fatal outcomes [11, 12]. A report suggested that elderly Asian patients with higher NIHSS scores might be more susceptible to extension of hemorrhage [12]. However, these reports are still preliminary, and there are no data from large-scale clinical trials.

Consideration also needs to be given to the fact that the studies with positive data are more easily published than those with negative data. Large-scale trials on IVT therapy with rt-PA after reversal of dabigatran with idarucizumab in acute ischemic stroke should be carried out with regard to unfavorable outcomes such as thrombotic events, hemorrhagic transformation, and mortality to further explore potential concerns regarding safety and efficacy of this treatment.

In our patient’s case, the significantly prolonged aPTT at the initial blood examination was immediately normalized, and the IVT therapy could be continued upon confirming dramatic reduction of the aPTT just after initiation of the rt-PA infusion, even though we had planned to terminate the IVT therapy if the aPTT was still prolonged [3]. We emphasize that, though this IVT therapy was carried out in an Asian woman just before the end of the recommended time limit, it was performed safely and resulted in a satisfactory outcome. We believe that this case report provides useful information regarding patients with acute ischemic stroke, including those with lacunar infarcts who are receiving dabigatran.

## Conclusion

We safely performed IVT therapy with rt-PA after reversal of dabigatran with idarucizumab in a patient with acute lacunar infarct. Treatment with rt-PA was administered in the emergency setting just before the end of the recommended time limit of IVT therapy.

## Data Availability

All data generated or analyzed during this study are included in this published article.

## Abbreviations

aPTT: Activated partial thromboplastin time
CT: Computed tomography
DOAC: Direct oral anticoagulant
IVT: Intravenous thrombolytic therapy
MR: Magnetic resonance
NIHSS: National Institutes of Health Stroke Scale
NVAF: Nonvalvular atrial fibrillation
rt-PA: Recombinant tissue plasminogen activator

## References

[1] Pollack CV Jr, Reilly PA, Eikelboom J, Glund S, Verhamme P, Bernstein RA, *et al.* Idarucizumab for dabigatran reversal. N Engl J Med. 2015;373:511–20. 10.1056/NEJMoa1502000 26095746

[2] Diener HC, Bernstein R, Butcher K, Campbell B, Cloud G, Davalos A, *et al.* Thrombolysis and thrombectomy in patients treated dabigatran with acute ischemic stroke: expert opinion. Int J Stroke. 2017;12:9–12. 10.1177/1747493016669849 27694315

[3] Toyoda K, Yamagami H, Koga M. Consensus guides on stroke thrombolysis for anticoagulated patients from Japan: application to other populations. J Stroke. 2018;20:321–31. 10.5853/jos.2018.01788 PMC6186921 30309227

[4] Schiele F, van Ryn J, Canada K, Newsome C, Sepulveda E, Park J, *et al.* A specific antidote for dabigatran: functional and structural characterization. Blood. 2013;121:3554–62. 10.1182/blood-2012-11-468207 23476049

[5] Kermer P, Eschenfelder CC, Diener HC, Grond M, Abdalla Y, Althaus K, *et al.* Antagonizing dabigatran by idarucizumab in cases of ischemic stroke or intracranial hemorrhage in Germany - a national case collection. Int J Stroke. 2017;12:383–91. 10.1177/1747493017701944 28494694

[6] Pikija S, Sztriha LK, Sebastian Mutzenbach J, Golaszewski SM, Sellner J. Idarucizumab in dabigatran-treated patients with acute ischemic stroke receiving alteplase: a systematic review of the available evidence. CNS Drugs. 2017;31:747–57. 10.1007/s40263-017-0460-x PMC5573762 28808918

[7] Giannandrea D, Caponi C, Mengoni A, Romoli M, Marando C, Gallina A, *et al.* Intravenous thrombolysis in stroke after dabigatran reversal with idarucizumab: case series and systematic review. J Neurol Neurosurg Psychiatry. 2019;90(5):619–23. 10.1136/jnnp-2018-318658 30032118

[8] Ohya Y, Makihara N, Wakisaka K, Morita T, Ago T, Kitazono T, *et al.* Thrombolytic therapy in severe cardioembolic stroke after reversal of dabigatran with idarucizumab: case report and literature review. J Stroke Cerebrovasc Dis. 2018;27:e128–31. 10.1016/j.jstrokecerebrovasdis.2018.02.025 29555397

[9] Kafke W, Kraft P. Intravenous thrombolysis after reversal of dabigatran by idarucizumab: a case report. Case Rep Neurol. 2016;8:140–4. 10.1159/000447531 PMC4945814 27482196

[10] Tse DM, Young L, Ranta A, Barber PA. Intravenous alteplase and endovascular clot retrieval following reversal of dabigatran with idarucizumab. J Neurol Neurosurg Psychiatry. 2018;89:549–50. 10.1136/jnnp-2017-316449 28986468

[11] Ng FC, Bice J, Rodda A, Lee-Archer M, Crompton DE. Adverse clinical outcomes after dabigatran reversal with idarucizumab to facilitate acute stroke thrombolysis. J Neurol. 2017;264:591–4. 10.1007/s00415-017-8410-6 28168523

[12] Tsai YT, Hsiao YJ, Tsai LK, Yen PS, Lin FY, Lu CH, *et al.* Idarucizumab-facilitated intravenous thrombolysis in acute stroke with dabigatran: two cases with hemorrhagic transformation. J Neurol Sci. 2018;15:155–7. 10.1016/j.jns.2018.03.021 29627012

